# “Submergence” of Western equine encephalitis virus: Evidence of positive selection argues against genetic drift and fitness reductions

**DOI:** 10.1371/journal.ppat.1008102

**Published:** 2020-02-06

**Authors:** Nicholas A. Bergren, Sherry Haller, Shannan L. Rossi, Robert L. Seymour, Jing Huang, Aaron L. Miller, Richard A. Bowen, Daniel A. Hartman, Aaron C. Brault, Scott C. Weaver

**Affiliations:** 1 Institute for Human Infections and Immunity, University of Texas Medical Branch, Galveston, Texas, United States of America; 2 Department of Pathology, University of Texas Medical Branch, Galveston, Texas, United States of America; 3 Department of Microbiology & Immunology, University of Texas Medical Branch, Galveston, Texas, United States of America; 4 Department of Pediatrics, University of Texas Medical Branch, Galveston, Texas, United States of America; 5 Department of Microbiology, Immunology, and Pathology, Colorado State University, Fort Collins, Colorado, United States of America; 6 Department of Biomedical Sciences, Colorado State University, Fort Collins, Colorado, United States of America; 7 Division of Vector-Borne Diseases, Centers for Disease Control and Prevention, Fort Collins, Colorado, United States of America; University of Glasgow, UNITED KINGDOM

## Abstract

Understanding the circumstances under which arboviruses emerge is critical for the development of targeted control and prevention strategies. This is highlighted by the emergence of chikungunya and Zika viruses in the New World. However, to comprehensively understand the ways in which viruses emerge and persist, factors influencing reductions in virus activity must also be understood. Western equine encephalitis virus (WEEV), which declined during the late 20^th^ century in apparent enzootic circulation as well as equine and human disease incidence, provides a unique case study on how reductions in virus activity can be understood by studying evolutionary trends and mechanisms. Previously, we showed using phylogenetics that during this period of decline, six amino acid residues appeared to be positively selected. To assess more directly the effect of these mutations, we utilized reverse genetics and competition fitness assays in the enzootic host and vector (house sparrows and *Culex tarsalis* mosquitoes). We observed that the mutations contemporary with reductions in WEEV circulation and disease that were non-conserved with respect to amino acid properties had a positive effect on enzootic fitness. We also assessed the effects of these mutations on virulence in the Syrian-Golden hamster model in relation to a general trend of increased virulence in older isolates. However, no change effect on virulence was observed based on these mutations. Thus, while WEEV apparently underwent positive selection for infection of enzootic hosts, residues associated with mammalian virulence were likely eliminated from the population by genetic drift or negative selection. These findings suggest that ecologic factors rather than fitness for natural transmission likely caused decreased levels of enzootic WEEV circulation during the late 20^th^ century.

## Author summary

Equally important to understanding how arboviruses emerge is understanding the conditions in which they experience reductions in activity. Western equine encephalitis virus (WEEV) provides a unique case study due to its reduction in equine and human incidence and wildlife transmission activity during the second half of the twentieth century. Despite those reductions, we identified six amino acid substitutions that appeared to increase fitness in avian hosts and/or mosquito vectors. We also found no effect of these mutations on mammalian virulence. Our results suggest that ecological factors likely explain the reduction in WEEV activity, even in the face of adaptive evolution.

## Introduction

Understanding the evolutionary and ecological circumstances in which arthropod-borne viruses (arboviruses) emerge, often into naïve geographical regions, is critical for the development of proactive, targeted control and prevention strategies. The need for this understanding has been highlighted by the recent emergence of chikungunya and Zika viruses in the Americas [1–3]. However, to develop a more complete understanding of the ways in which viruses emerge, the factors surrounding reductions in virus activity, or “submergence,” must also be studied. Western equine encephalitis virus (WEEV) provides a unique case study in such submergence and an opportunity to study the evolutionary factors associated with the dramatic reduction in human and equine cases during recent decades.

**Fig 1:**
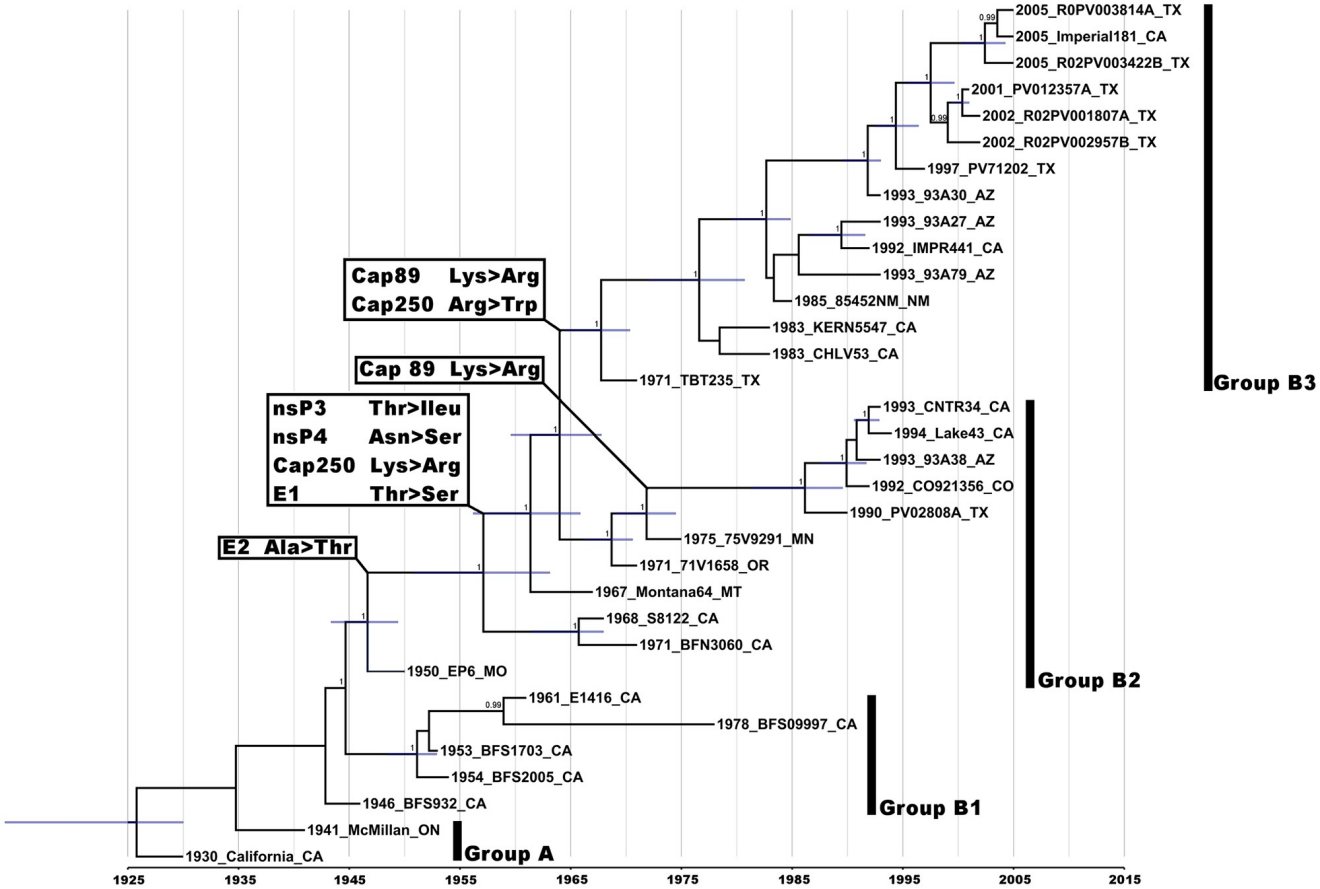
Maximum clade credibility tree based on thirty-three WEEV genomes. Numbers at nodes indicate posterior probabilities of ≥ 0.9. Bars at nodes indicate 95% confidence intervals of divergence dates, and the x-axis represents time in years. The four distinct lineages, groups A and B1 through B3, are indicated. Nonsynonymous synapomorphic mutations are indicated on the tree based on their identified node of occurrence. Taxon/tip labels include year of isolation, strain name, and state where the virus was isolated. Figure originally published by Bergren et al. (2014) Western Equine Encephalitis Virus: Evolutionary Analysis of a Declining Alphavirus Based on Complete Genome Sequences. Journal of Virology. 2014;88(16):9260–7. doi: 10.1128/jvi.01463-14. https://jvi.asm.org/content/88/16/9260.

WEEV is an arbovirus in the genus *Alphavirus*, family *Togaviridae* [4]. The WEEV genome consists of a single-stranded, positive-sense RNA, approximately 11.5kb in length, with a 5’ cap and polyadenylated tail. The genomic RNA encodes four nonstructural proteins (nsP1-4) and a subgenomic RNA encodes the structural proteins: capsid, E1-3 and 6K/TF [5, 6]. WEEV is found in both North and South America and is a member of the Western equine encephalitis alphavirus serocomplex along with Sindbis and Highlands J viruses, among others. WEEV occurs in several lineages, some of which appear to be restricted to South America while others occur in both North and South America. Two primary genetic lineages (Groups A and B) have circulated in North America, with Group B having three sublineages (B1-B3) [7, 8]. Group A strains were isolated between 1930 and 1941, Group B1 strains between 1946 and 1961, Group B2 strains between 1950 and 1993, and Group B3 strains from 1971 to the present [7]. Fig 1 details this temporally structured phylogeny with a maximum clade credibility tree based on the complete open reading frames of thirty-three WEEV isolates [7].

WEEV is transmitted among avian vertebrate hosts by mosquito vectors. Its circulation in North America has been well characterized; the annual transmission cycle can be divided seasonally, with the virus amplifying in the spring, maintained during the summer, declining in the fall, and quiescent during the winter [9]. The principal enzootic host and vector for WEEV are house sparrows (*Passer domesticus*) (HOSPs) and *Culex* (*Culex*) *tarsalis* Coquillett mosquitoes, respectively [9]. During years of high enzootic activity, WEEV can also infect a variety of mammals and initiate an independent mammal/*Aedes* spp. cycle [10–13]. The ecology of WEEV in South America includes *Aedes* (*Ochlerotatus*) *albifasciatus* (Macquart) as a probable enzootic vector with various birds and mammals acting as reservoir/amplification hosts [14, 15].

WEEV is the etiologic agent of western equine encephalitis or encephalomyelitis (WEE) [16]. Encephalitis occurs in both humans and horses, both of which are historically considered to be dead-end hosts (i.e. viremia does not reach levels sufficient to infect potential mosquito vectors) [9]. However, some studies have shown that equids such as ponies and burros develop viremias capable of infecting mosquitoes [17–19]. Thus, the role of equids as amplification hosts remains uncertain.

Human WEE can range from a mild febrile illness to overt encephalitis, leading to coma or death [9]. The case-fatality rate in humans ranges from 3 to 15% depending upon the specific epizootic/epidemic event, and severity is skewed toward infants, young children, and the elderly [9, 20]. The equine case-fatality rate ranges from 10 to 50% [9]. Although no licensed vaccine is available for human use, formalin-inactivated vaccines have been available for equids since the late 1940s and are generally administered annually [21].

WEEV was an important human and veterinary pathogen in the early-to-mid-20^th^ century; however, as the 20^th^ century proceeded, human WEE incidence in the U.S. and Canada declined precipitously with the last case in North America reported in 1998 [9, 22]. Equine WEE has also declined with the last substantial epizootic occurring in 1975 in North Dakota and Manitoba, and smaller, sporadic equine epizootics occurring into the 1990s [23, 24]. Additionally, the rates of WEEV-positive mosquito pools and seropositive birds detected during routine surveillance in California have declined [25]. The latest identification of WEEV in its enzootic cycle was a positive mosquito pool collected in 2013 in Clark County, Nevada [26]. However, it is worth noting that WEEV has recently been removed from the Center for Disease Control and Prevention’s (CDC’s) ArboNET surveillance system. A small scale WEEV surveillance study was carried out in Larimer County, Colorado during the summer for 2017 and yielded no detection of WEEV in *C*. *tarsalis* or *A*. *melanimon* pools further, emphasizing the precipitous decline of WEEV in enzootic vectors [27].

Despite the drastic reduction in human and equine incidence and enzootic activity, the WEEV genome has changed little for an arboviral RNA genome since 1930, with only a maximum of 3.7% nucleotide sequence divergence among North American isolates collected over a period of more than 70 years [7]. Interestingly, recent WEEV isolates in general appear to be less virulent in murine models than strains isolated in the early 20^th^ century [28, 29]. A glutamine residue on position 214 of the E2 glycoprotein, present throughout the Group A lineage, is a neurovirulence determinant. When the glutamine was replaced with arginine (the residue present at the position in the Group B viruses) on WEEV/McMillan strain, a prototypical Group A virus, murine neurovirulence was reduced [30]. Conversely, when the reciprocal mutation was engineered into strain WEEV/IMP181 (a prototypical, contemporary Group B virus) neurovirulence was not increased [30]. However, the murine virulence of WEEV strains lacking this mutation indicates that additional virulence factors exist [28]. Moreover, virulence determinant conclusions are further confounded due to the possibility that the E2-Q214R substitution arose as a result of the extensive use of infant mice to passage viruses isolated in the 1930s and 40s [7].

In terms of fitness for circulation in the enzootic cycle, Group A viruses seem to generally be less fit than Group B viruses in mosquitoes [30], and changes in enzootic fitness within Group B are difficult to determine within the present literature. For example, no discernable trend could be determined when comparing the viremia titers of representative WEEV isolates from each decade from the 1950s through the 2000s in white crowned and house sparrows. For example, strains BFS1703 and COA592, representing the 1950s and 1990s, respectively, both had elevated virus titers as compared to the other strains tested [31].

Our previous phylogenetic studies indicated a decline in the WEEV population size during the late 20^th^ century, suggesting that genetic drift, possibly accompanied by Muller’s ratchet (fitness declines following repeated population bottlenecks in the absence of efficient recombination to restore random mutations to the original sequence) [32], could have resulted in fitness declines for enzootic circulation. Furthermore, we found that between 1950 and 1970, six nonsynonymous mutations arose and became fixed in all currently circulating Group B viruses (Fig 1, Table 1) [7]. These mutations were informatically suggested to have been positively selected (though none reached statistical significance as determined by the models used), and thus could have played a role in putative WEEV fitness changes during the 20^th^ century. These two findings are somewhat contradictory as a population that is declining in fitness through constant reductions in population size and the accumulation of deleterious mutations through drift should not have much opportunity to undergo positive selection. Thus, the hypothesis emerged that the six nonsynonymous mutations were positively selected based on thereby beneficial effect on enzootic fitness, but they did not exert enough of an advantage to counteract the reduced population size and drift that accompanied reduced WEEV transmission.

**Table 1:** Nonsynonymous synapomorphic mutations that contribute to the definition of WEEV lineages.

Protein	Change in Amino Acid	Amino Acid Position	Mutation	Codon Position
nsP3	Thr > Ileu	152	C > T	2
nsP4	Asn > Ser	602	A > G	2
Capsid	Lys > Arg	89	A > G	2
Capsid	Lys > Trp	250	A > T	1
			A > G	2
E2	Ala > Thr	23	G > A	1
E1	Thr > Ser	374	A > T	1

To assess the phenotypes of these six putatively positively selected mutations, we tested their effect on enzootic host and vector fitness as well as mammalian virulence. To accurately and sensitively assess enzootic host and vector fitness, we utilized competition assays in *C*. *tarsalis* and HOSPs. Changes in the ratios of two competing viruses, assessed through pyrosequencing, allow for reproducible, internally controlled, highly sensitive comparisons of fitness with limited numbers of infections [33–35]. We also assessed the effect of these six mutations on mammalian virulence using the Syrian golden hamster model.

## Results

### Viruses

**Fig 2:**
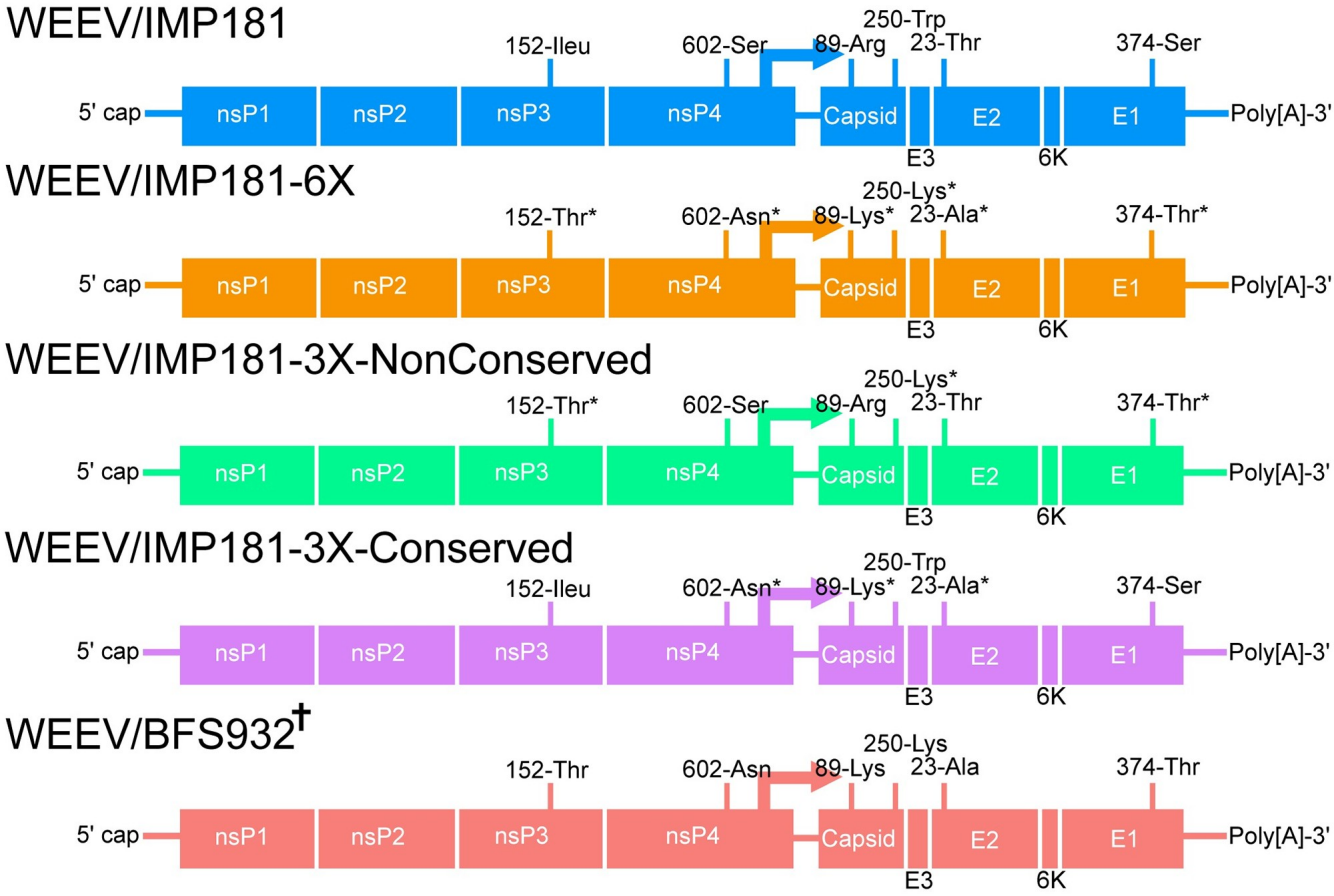
Diagram of viruses used throughout the study and their relevant mutations. Amino acid residues listed on WEEV/IMP181 and WEEV/BFS932 reflect unaltered amino acids at sites of interest. Amino acids with an asterisk reflect changes made to derive the specific construct. ^†^Note that WEEV/BFS932 is not derived from an infectious clone but is a plaque purified isolate.

For reverse genetic experiments we utilized the WEEV/Imperial181 (IMP181) infectious cDNA clone, derived from a strain isolated in 2005 in a pool of *C*. *tarsalis* adult females collected in Imperial Co., CA; this strain belongs to Group B3, and is relatively avirulent in murine models [29, 30]. We used this clone as a backbone to revert six of the critical, putatively positively selected substitutions described above, yielding IMP181-6X (ancestral amino acids at these six positions). Two additional clones were also constructed on the IMP181 backbone, each with three of these six mutations. We divided the mutations based on conservative/non-conservative amino acid changes and the predicted location of the amino acid residues on the folded E2 protein, to yield IMP181-3X-NonConserved (containing the ancestral mutations nsP3 152Thr, capsid 250Lys, and E1 374Thr) and IMP181-3X-Conserved (containing the ancestral mutations nsP4 602Asn, capsid 89Lys and E2 23Ala) (Fig 2). While the mutation in E1 is considered conservative, we included it in the non-conservative construct due its predicted location at the E1-E2 interface, which could affect the stability of the envelope glycoprotein dimer on the virion spike [7]. As a control throughout the experiments, to account for potential epistatically interacting mutations not included in the mutated IMP181 constructs, we also utilized strain WEEV/BFS932 (BFS932) a Group B1, virulent, low passage isolate from 1946. This strain contains all the 6 ancestral amino acids tested and does not contain the putatively mouse-adapted neurovirulence mutation at position 214 in E2 that is characteristic of Group A viruses [30].

### Culex tarsalis competition assays

**Fig 3:**
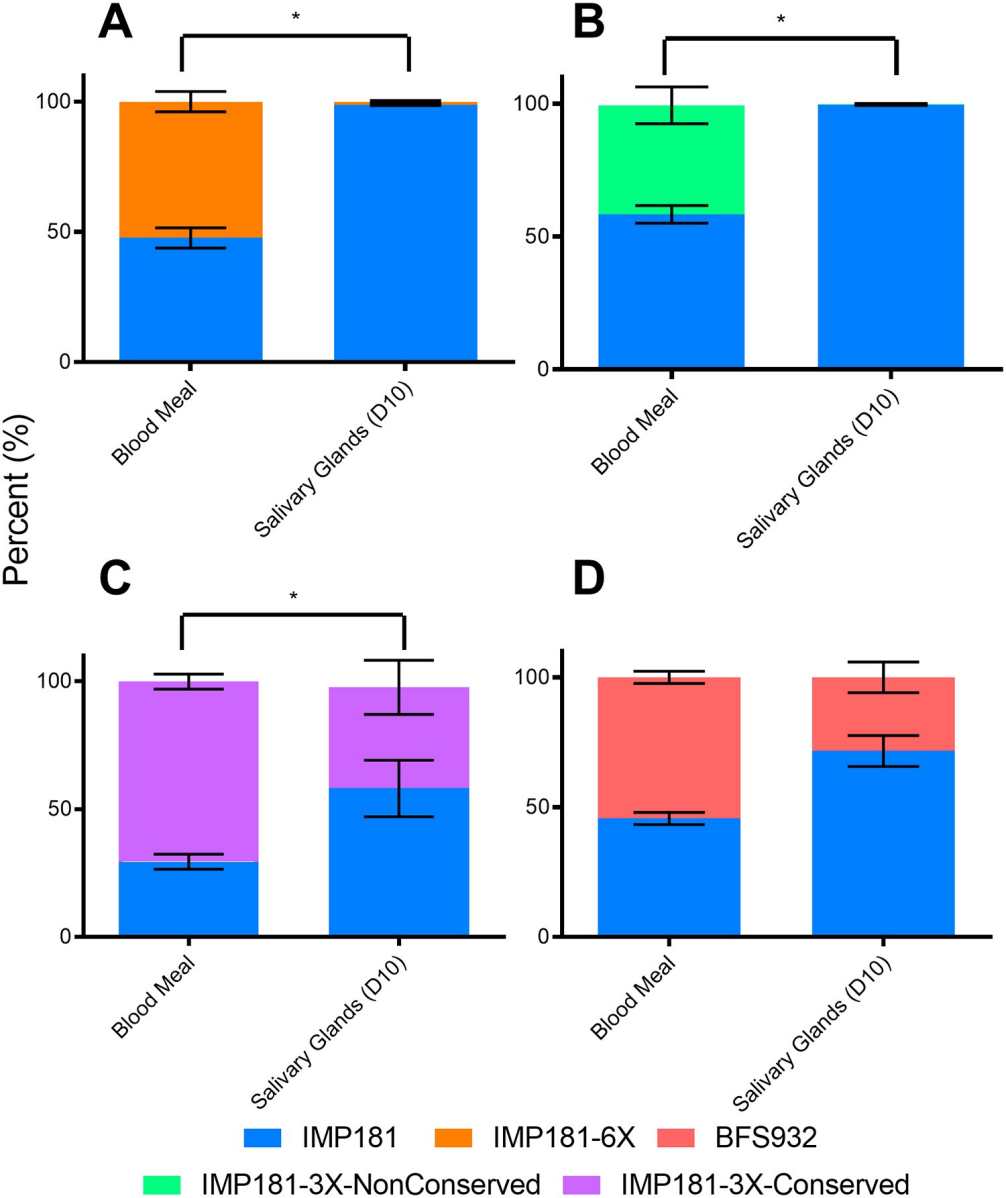
Competition assays in *C*. *tarsalis* showing the ratios of virus in the blood meal and salivary glands on day 10 post-blood meal. Mean and standard error of replicates (3 replicates with n = 5 per replicate) in assays competing A) IMP181 v. IMP181-6X (p-value 0.0005318); B) IMP181 v. IMP181-3X-NonConservative (p-value 0.0005303); C) IMP181 v. IMP181-3X-Conservative (p-value 0.01245); and D) IMP181 v. BFS932 (p-value 0.08105). *Indicates significant (p ≤ 0.05) change in virus ratio as determined by Wilcoxon test.

*Culex tarsalis* is the primary enzootic vector for WEEV [9]. Female mosquitoes from the Kern BFS colony [36], were allowed to feed on artificial blood meals containing 6 log_10_ PFU/ml of competing virus mixtures, each in triplicate, including IMP181/BFS932, IMP181/IMP181-6X, IMP181/IMP181-3X-NonConservative, and IMP181/IMP181-3X-Conservative mixtures. Aliquots of the artificial blood meals were analyzed by pyrosequencing and compared to mosquito salivary gland-derived virus [determined to be infected by a cytopathic effect (CPE) assay] from day 10 post-bloodmeal to determine changes in the virus ratio during infection, replication and dissemination in the vector; 95% of these salivary glands were WEEV-positive (S1 Fig). When competed against IMP181-6X, IMP181-3X-NonConservative, and IMP1813X-Conservative the parental IMP181 was more fit by a significant margin (Fig 3A and 3B). This finding was corroborated by IMP181 outcompeting the older BFS932 strain (Fig 3D), though by a smaller margin which was not significant.

### House sparrow competition assays

**Fig 4:**
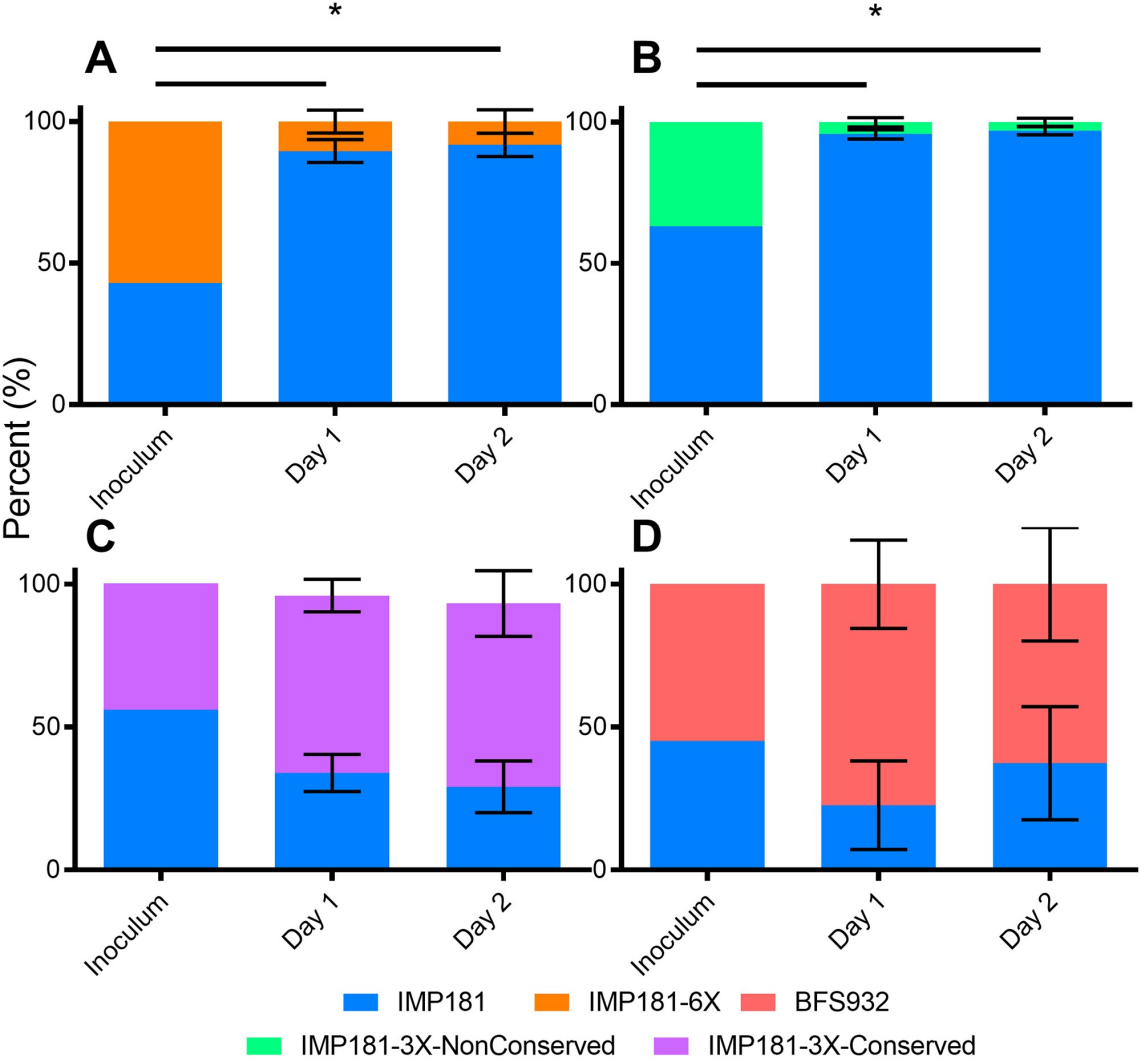
Competition assays in HOSPs showing the ratios of virus in the inoculum and serum on days 1 and 2 post-infection. Mean and standard error in assays competing A) IMP181 v. IMP181-6X (p-value 0.02201); B) IMP181 v. IMP181-3X-NonConservative (p-value 0.01991); C) IMP181 v. IMP181-3X-Conservative (p-value 0.25); and D) IMP181 v. BFS932 (p-value 1.0) (n = 7 per group). * Indicates significant (p ≤ 0.05) change in virus ratio as determined by Wilcoxon test.

HOSPs are a natural enzootic amplification/reservoir host for WEEV; they develop a high-titer, short-lived viremia that peaks on day 1 post-infection, then falls precipitously, generally without detectable morbidity [31]. HOSPs were subcutaneously inoculated with virus mixtures (listed above) containing 3 log_10_ PFU of each competitor. Aliquots of the inoculum was analyzed by pyrosequencing and compared to serum samples from day 1 and 2 post-infection to determine changes in the competitor virus ratios. When IMP181-6X and IMP181-3X-NonConservative were competed against IMP181, the IMP181’s frequency was significantly greater than IMP181-3X-NonConservative on days 1 and 2 post-infection, indicating its competitive advantage (Fig 4A and 4B). No significant fitness difference was determined when IMP181 was competed against IMP181-3X-Conservative (Fig 4C). Additionally, the assay competing BFS932 against IMP181 did not indicate a clear fitness difference, indicating that these strains are similarly fit for HOSP amplification (Fig 4D). This result is likely due to the presence of other residues in BFS932 that counteract the three non-conservative substitutions for replication in HOSPs, independent of the six mutations tested.

### Hamster virulence assays

To elucidate possible virulence effects of the six mutations, we compared the relative virulence of IMP181, BFS932, and IMP181-6X. The rationale for using BFS932 as a control was to determine if IMP181-6X was producing an infection more similar to an ancestral, virulent strain of WEEV that contained the same 6 ancestral amino acids, but also many other differences. Female hamsters, 5-6-weeks-old, were infected intraperitoneally (IP) with 4 log_10_ PFU. Weights and morbidity were monitored daily and viremia was measured every two days for each animal in staggered cohorts. Additionally, three hamsters per group were perfused with PBS on days 2–5 to assess the viral load in their major organs without contamination from viremic blood, and to assess any histopathologic changes.

**Fig 5:**
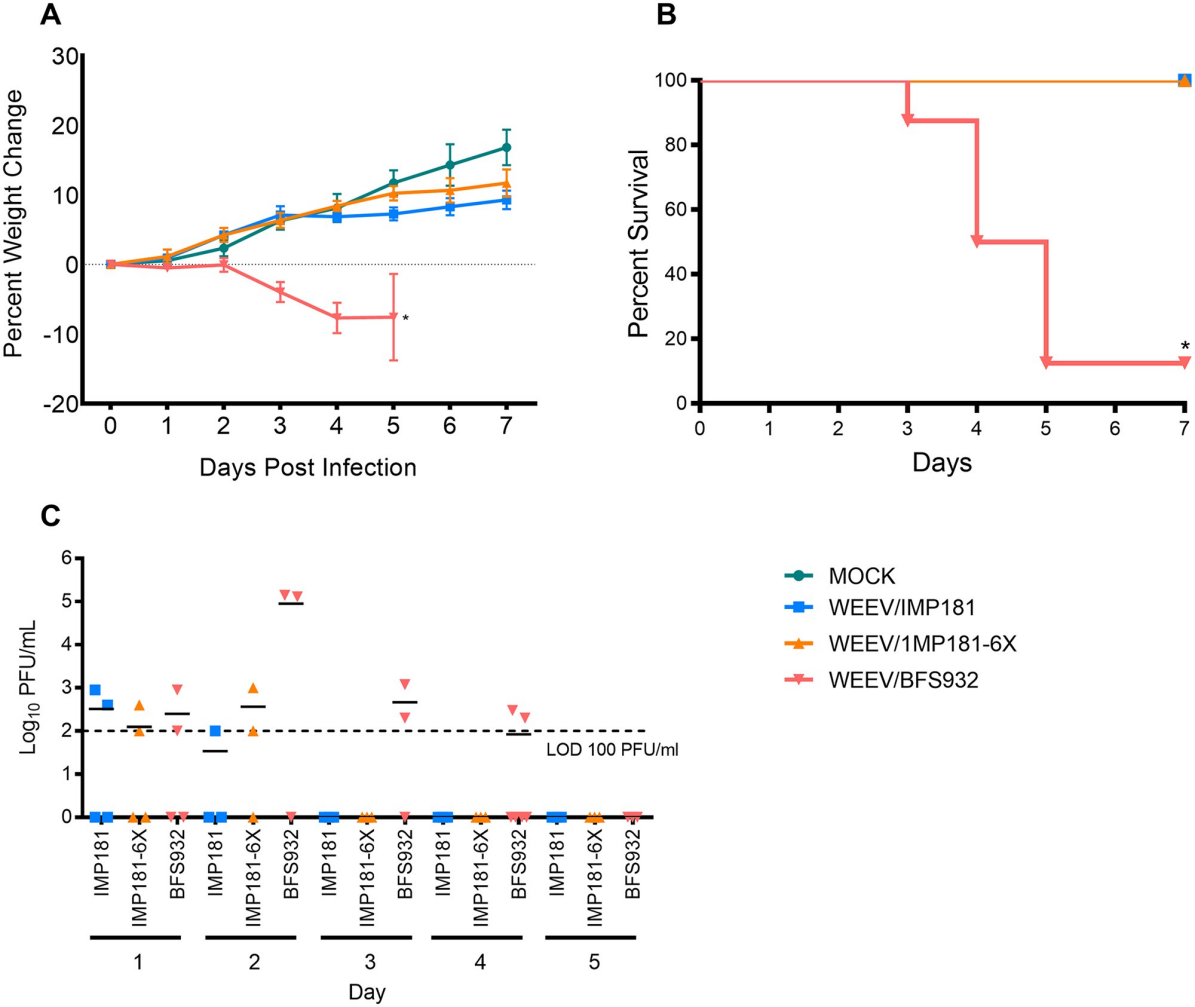
Weight, survival, and viremia in 5–6 week old Syrian golden hamsters following infection with IMP181, IMP181-6X, and BFS932. Panels show A) weight; B) survival; and C) viremia. *Indicates statistical significance (p ≤ 0.05).

**Fig 6:**
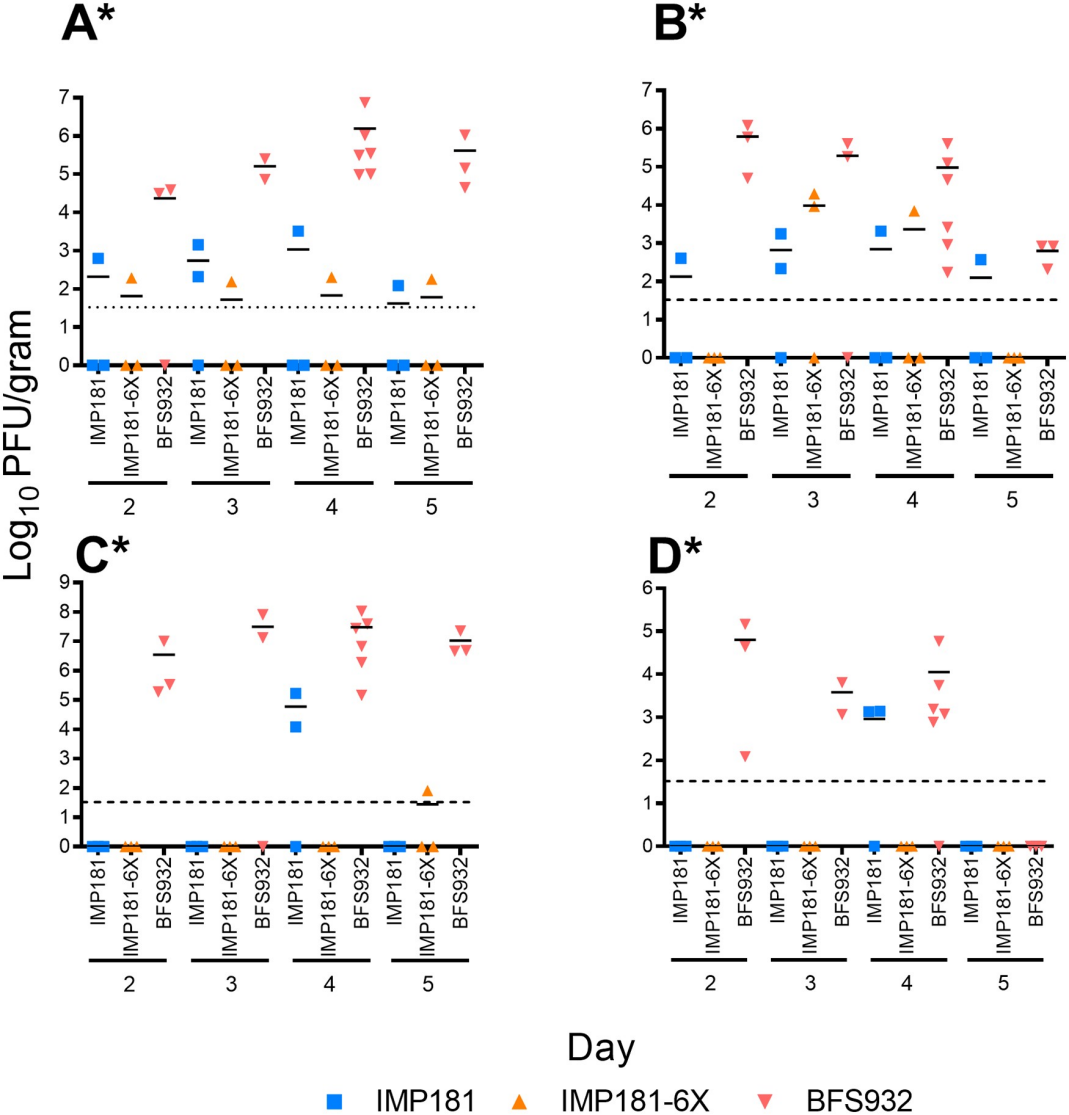
Viral burden in 5–6 week old Syrian golden hamsters that show significant differences following infection with IMP181, IMP181-6X, and BFS932. Panels show viral burden in the (A) brain, (B) heart; (C) muscle; (D) kidney. *Indicates statistical significance (p ≤ 0.05).

**Fig 7:**
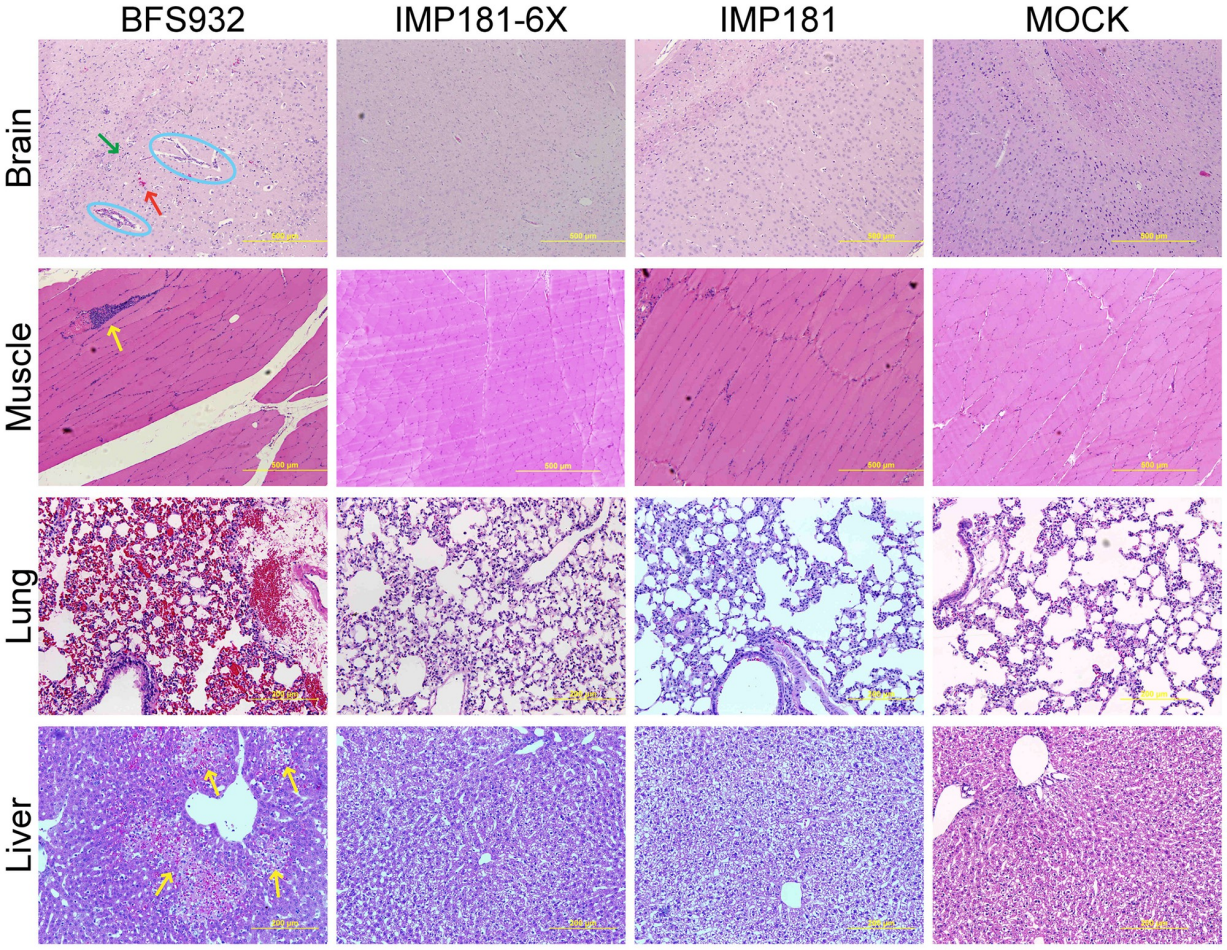
Differences in histopathology during peak disease of 5–6 week old Syrian golden hamsters infected with IMP181, IMP181-6X, and BFS932. Brain and muscle images at 10X; perivascular cuffing, hemorrhage, and mononuclear infiltration marked with blue circles, red arrows, and green arrows, respectively. Yellow arrows on muscle slides indicate myositis. Liver and Lung images taken at 20X; yellow arrows on liver slides indicate foci of necrosis. All BFS932 images are from day 4 post-infection. IMP181-6X, IMP181, and MOCK were taken at day 5 post-infection.

Hamsters inoculated with IMP181 tolerated infection well with 100% survival and no significant difference in weight compared to sham-infected animals. IMP181-6X exhibited the same pattern of virulence as compared to IMP181. In contrast, all but one BFS932-infected animal became moribund (Fig 5A and 5B), with significantly higher viral loads in the brain, heart, muscle, and kidney as compared to the other viruses (Fig 6). No significant differences were determined for the spleen and only IMP181-6X was significantly different from BFS932 for lung and liver (S2 Fig). Brain histology showed no lesions in IMP181 or IMP181-6X-infected hamsters, whereas signs of severe encephalitis were present after BFS932 infection, including perivascular cuffing, mononuclear infiltrates, and hemorrhage (Fig 7 & S3 Fig). Mild myocarditis was found in all groups (S4 Fig) and mild myositis was observed only in BFS932-infected hamsters (Fig 7). Several foci of necrosis were found in the livers of BFS932-inoculated hamsters, while IMP181 and IMP181-6X livers were indistinguishable from those of sham-infected animals (Fig 7).

## Discussion

Although WEEV caused many major outbreaks of human and equine encephalitis in western North America throughout the early- to mid-20^th^ century, the late-20^th^ century was characterized by a marked reduction in WEEV activity with the last human case documented in 1998 [22], and considerably reduced numbers of infected mosquito pools identified in longitudinal surveillance programs [25, 26]. We experimentally tested our hypothesis, based on phylogenetic findings, that six nonsynonymous mutations were positively selected because of a beneficial effect on enzootic fitness, but that these mutations did not exert enough of an advantage to counteract reduced WEEV population sizes, possibly due to ecologic changes [7].

To reliably determine the fitness effect of the six nonsynonymous mutations, competition assays were conducted in enzootic hosts and vectors. We chose to use competition assays to capitalize upon their high degree of sensitivity and efficiency as demonstrated previously for several viruses including alphaviruses [37–43]. In a competition assay, a virus with a small competitive advantage over its competitor virus will undergo an increase in its frequency in the population that is easily measured using molecular methods [37–39]. Historically, such mutations would have been assessed by standard vector and host competence experiments where each individual virus would be used to infect large numbers of hosts or vectors, and virus infection and replication would be assessed in each individual; differences between infection rates and/or virus titers would then be assessed statistically. This approach was previously applied to different WEEV stains in an attempt to explain a potential reduction in enzootic WEEV activity with limited success [31]. Therefore, we employed the more sensitive competition method to more accurately discriminate small fitness differences. The competition technique avoids variation in assays of individual host titers because only the ratio of the two viruses is measured. The competitions are also internally controlled so variation in individual mosquito or bird susceptibility does not reduce the power of the experiments. Both of these advantages also increase efficiency by reducing the numbers of animals required. Also, we focused only on *in vivo* experiments because previous studies with other alphaviruses indicate that *in vitro* fitness infrequently correlates with *in vivo* findings [40, 42, 44–46].

Our choice of competition assay also mitigated the limitation of our use of the Bakersfield colony of *Cx*. *tarsalis*, which is highly competent for WEEV in part due to its colonization since 1958 [36, 47]; note our extremely high rates of infected salivary glands (S1 Fig). Thus, standard vector competence experiments with this colonized mosquito using individual mutants would likely have yielded results difficult to distinguish statistically. While it is unlikely that vector competence of this colony could have changed with respect to individual WEEV mutations, additional studies with wild or recently colonized mosquitoes should be undertaken.

Our results show that IMP181 outcompeted both IMP181-6X and IMP181-3X-Nonconservative in *C*. *tarsalis* and HOSPs. Thus, the three non-conservative amino acid substitutions that occurred coincident with the reduction in WEE during the second half of the 20^th^ century conferred a fitness advantage. The lack of a significant fitness difference between IMP181 and BFS932, which differ in the same mutations, in *C*. *tarsalis* and HOSPs could reflect genetic difference between these strains other than the 6X, and possibly epistatic, that confer enzootic fitness. Indeed, when IMP181 and BFS932 are analyzed for non-synonymous mutations there are 26 and 23 non-synonymous mutations present in their non-structural and structural ORFs, respectively. Competition assays in *C*. *tarsalis* and HOSPs indicate that the non-conservative, nonsynonymous synapomorphic mutations that were fixed in the WEEV populations during the mid-20^th^ century conferred fitness gains for transmission by *C*. *tarsalis* mosquitoes, consistent with their positive selection.

Our hamster experiment showed IMP181-6X and IMP181 produced no discernable disease, indicating that these six historic mutations have no effect on this model for mammalian virulence, while BFS932 was significantly more virulent. This finding suggests that unidentified mutations in BFS932 affecting virulence were eliminated from the WEEV the population via negative selection or drift. Future experiments using chimeras between BFS932 and IMP181 should be used as a first step to identify these putative virulence determinants.

Our results argue against a sustained, dominant Muller’s Ratchet or drift effect to explain the reduction in WEEV circulation during the late 20^th^ century. The prolonged fixation of deleterious mutations in the population would be inconsistent with the fitness differences in *C*. *tarsalis* and HOSPs that were indicated by our competition of the recent IMP181 strain with IMP181-6X. Furthermore, the competitions between the natural isolates IMP181 and BFS932 showed that IMP181 is likely more fit in *C*. *tarsalis* and at least equally fit in HOSPs. Our previous phylogenetic findings indicating that the WEEV population is in decline and experiencing purifying selection cannot be completely discounted. However, purifying selection is typically the dominant form of selection identified in phylogenetic studies of many arboviruses, inducing those undergoing emergence [48–51]. This suggests that the presence of purifying selection is of limited value in explaining arbovirus population declines.

Ecological factors accounting for WEEV’s population decline also deserve consideration. One is that equids (possibly juveniles) at one time participated in epizootic transmission of WEEV. However, even if equids served as amplification hosts during the past, they were likely eliminated from this role via their vaccination beginning in the 1940s, combined with their drastic population reductions during the 20^th^ century as a result of mechanized agriculture [52]. This may have relaxed selection for WEEV mammalian amplification determinants, resulting in elimination of virulence determinants by purifying selection if they did not contribute to fitness in avian or mosquito hosts. This hypothesis is supported by evidence that donkeys and ponies can develop viremias high enough to infect a mosquito vector [17, 18].

The limited scope of our experiments did not allow us to address the following issues possibly related to the decline in WEEV circulation and disease incidence: 1) did the vector competence of *C*. *tarsalis* change over time? 2) what is the vector competence for individual virus strains within each WEEV lineage? 3) what is the specific adaptive pathway taken by WEEV during the 20^th^ century? and 4) do non-synapomorphic (not shared by all descendants) mutations that we did not examine interact epistatically with the nonsynonymous synapomorphic (shared by all descendants) residues that we tested? These questions will require additional, extensive experiments.

Taken together, our results support the hypothesis that WEEV, despite undergoing a population decline, underwent fitness gains for enzootic transmission during the 20^th^ century. Previous phylogenetic analyses demonstrated that, during the 20^th^ century, WEEV followed an influenza virus-like pattern of lineage emergence and purification [7]. This pattern ultimately resulted in the continuation only of Group B3 and a subsequent, putative reduction in population size. Additionally, six putatively positively selected residues have been identified [7]. Using these phylogenetic findings to guide our reverse genetic experiments, we found evidence that the three non-conservative nonsynonymous synapomorphic mutations that were fixed in the WEEV population during the 20^th^ century were positively selected for enzootic transmission. The evidence of positive selection is a bit surprising in the context of our previous phylogenetic findings indicating a decline in the WEEV population and a possible sustained Muller’s Ratchet effect [7, 25]. However, it is important to note that the positively selected mutations arose during an earlier period of elevated population levels [7]. Other studies also indicate that non-conservative mutations, selected positively, can have profound effects on alphaviruses [40–43, 53, 54]. Our findings therefore support the idea that WEEV population reductions were the result of external factors that reduced enzootic circulation. While the specific external factor(s) remains unknown, the efficient transmission by *C*. *tarsalis* of West Nile virus in western North America since 2002, at the time of WEEV decline, suggest that reductions in vector populations are not an explanation.

In summary, our results provide a unique perspective on the evolution of WEEV through the 20^th^ century, suggesting that shifts in arbovirus ecology can have profound impacts on human and equine exposure even in in the face of adaptive evolution. The specific ecological circumstances that precipitated this putative downward evolutionary trajectory remain cryptic. The potential role of equids in previous WEEV amplification and the effect of equine vaccination on enzootic circulation also deserve further study [21, 52]. Finally, our results underscore that poorly understood effects on arbovirus circulation, which may carry no genetic signature in phylogenetic analyses, can have profound effects on disease incidence.

## Materials and methods

### Cell culture

Vero cells (ATCC CCL-81, American Type Culture Collection, Manassas, VA) were maintained in DMEM (Dulbecco’s Modified Eagle Medium) (Gibco, Carlsbad, CA), 10% (vol/vol) fetal bovine serum (FBS), 1% (vol/vol) 100X Non-Essential Amino Acids (Sigma-Aldrich), 1% (vol/vol) Sodium Pyruvate (100mM) (Gibco) and 1% (vol/vol) Penn-Strep (Penicillin & Streptomycin) (5,000 U/ml) (Gibco). Cells were incubated at 37°C with 5% CO_2_ in a humidified incubator.

### Viral infectious clone plasmids

Infectious clones, containing all or half of the mutations previously identified (Table 1), were generated via site directed mutagenesis. Briefly, WEEV/IMP181-6X contains all mutations listed in Table 1. The clones with half of the mutations were divided into two clones based on their amino acid change or the relevance of their location on the protein. Changes that conferred non-conservative amino acid changes or were located on a relevant functional area of the E1 glycoprotein were included on one clone, named IMP181-3X-NonConservative. Conversely, changes that conferred a conservative amino acid change or had a location on the protein of unknown importance were included on the other clone, named IMP181-3X-Conservative. The specific mutations each clone contains can be found by referring to Table 1 and Fig 2. E1 and E2 mutations were determined by their location on their respective protein [7].

### Plasmid purification and in vitro transcription

Stocks of infectious clones were generated, sequences were verified, and RNA was transcribed as previously described [55]. RNA was stored at -80°C for no more than 24 hours prior to electroporation.

### Virus stocks

Stocks of WEEV/BFS932 (BFS932) were generated by picking a plaque of the virus and subsequently using the picked plaque to infect Vero cells and generate a stock of virus, the sequence of the resulting stock was verified [56]. Electroporation of *in vitro* transcribed RNAs was used to generate stocks of all other wild-type and mutant WEEV strains, and the sequence of output virus was verified [55]. Stock titers were determined by plaque assay as previously described [57].

### Competition assays

In order to have a better understanding of the effect of the discovered mutations, competition assays between mutant virus and wild-type IMP181 were conducted in various models with the appropriate controls, with virus ratios determined by pyrosequencing. One important note concerning the competition assays we conducted was that we mixed by infectious units (PFUs) and measured the ratio of the viruses by relative genome equivalents. The PFU:genome ratio was fairly consistent between all of our constructs, as indicated by the inoculum and blood meals measured for the HOSPs and mosquitoes.

#### Mosquitoes

The Bakersfield colony of *C*. *tarsalis*, isolated in 1958, was used for the following studies [36]. Triplicate mixtures of viruses were diluted to 7 log_10_ PFU/ml mixed at a 1:1 ratio based on plaque forming units (PFU). Proper titers were verified by conducting plaque assays on each diluted virus before they were mixed. Virus mixtures were subsequently mixed at a ratio of 1:1 with artificial blood meal formulated with PBS-washed chicken blood (Colorado Serum Company, Denver, CO) as previously described [48]. Aliquots of blood meals were collected to provide a comparison to the salivary glands collected. After 10-days-post blood meal salivary glands were dissected (5 per replicate on day 10-post blood meal) and placed in a 2ml Eppendorf Safe Lock tube (Hamburg, Germany) with 250μl DMEM (supplemented as described above) with 25 mg/ml Amphotericin B and a steel ball bearing. Samples were stored at -80°C.

#### House sparrows

HOSPs were caught using mist nets in Larimer County, CO and transferred to the ABSL3 at Colorado State University (CSU). Previous exposure to WEEV was determined by hemagglutination-inhibition (HI) and only naïve HOSPs were used. Mixtures of viruses were diluted to 4 log_10_ PFU/ml and mixed at a 1:1 ratio. HOSPs were subcutaneously inoculated (SC) with 100μl diluted virus mixture. Titers of viruses before mixing were taken to verify proper concentrations. Also, an aliquot of the inoculum was reserved for testing the initial ratio of viruses. HOSPs were bled on days 1 and 2-post infection via the jugular vein. Serum was collected from blood via centrifugation at 12,000 rcf for 5 minutes, and stored at -80°C until analysis. Pyrosequencing analysis was conducted as described below.

#### CPE assays

Prior to pyrosequencing analysis, virus positive samples were determined by CPE (cytopathic effect) assay as previously described [28].

#### Sample preparation and Nucleic acid extraction

Aliquots of mosquito lysates and serum samples were prepared in Roche (Basel, Switzerland) external lysis buffer IVD (200μl) and deposited into individual wells of 96 deep-well processing plates (Roche Applied Science). Nucleic acids were subsequently extracted in high-throughput fashion using a Magna Pure 96 instrument employing large-volume Cellular RNA extraction kits (Roche) according to the manufacturer’s protocol for fresh/frozen biological samples. After extraction, a portion of the RNA was immediately converted to cDNA and the remaining material was archived at -80°C.

#### Reverse transcriptase

cDNA was synthesized from extracted RNA using an iScript synthesis kit (Bio-Rad, Hercules, CA). 40μl reactions were assembled in 96 well PCR plates (Thermofisher Scientific) through the addition of 8μl iScript reaction mix, 2μl reverse transcriptase and 30μl of extracted RNA. RT was completed using a Bio-Rad C1000 thermocycler using the following protocol: 1) 25°C, 1.5 min; 2) 42°C, 30 min; 3) 85°C, 5 min; 4) indefinite hold at 4°C. Generated cDNA was analyzed immediately then stored at -20°C.

#### Pyrosequencing

WEEV pyrosequencing (a sequencing by synthesis technology) was performed using PCR-based pyrosequencing. Primers were designed using Pyromark Assay Design 2.0 software for SNP analysis. Initial PCR was carried out as follows: 12.5μl of iQ supermix (Bio-Rad, Hercules, CA) was mixed within a 25μl PCR reaction containing 200nM of both forward and reverse primers, 3μl cDNA and nuclease-free water. The specific primers used in each assay and the sequences analyzed are listed on Table 2. Thermocycling was completed using a Bio-Rad C1000 thermocycler using the following protocol: 1) 95°C, 3.0 min; 2) 95°C, 30s; 3) 60°C, 30s; 4) 72°C, 30s repeat 50x; 5) 72°C, 2 min; 6) indefinite hold at 4°C. Generated biotinlyated PCR products were pyrosequenced using PyroMark Gold reagents on a PyroMark Q96 ID platform according to the manufacturers’ instructions (Qiagen). Sequencing primers were diluted to a final concentration of 0.3μM, in combination with SNP mode setting, were used to perform pyrosequencing.

**Table 2:** Primers and viral sequence analyzed in pyrosequencing assay.

Pyrosequencing Assay	nsP3 Based	nsP4 Based
Primers (Forward/Reverse)	GCCGATGTCACCATATATTGCTT	Biotin-CGTTAGCCGAAAGCGTTAAGAACT
	Biotin-CATCCAGTATTTCGACGCTTTCTT	TTTAGGTCAGCCGTAGAGGGTGAT
Sequencing Primers	AATGGGAGACCAGGA	CGTAGAGGGTGATTGG
Sequence Analyzed	TAAYCGAGGCCATTCACCGCAAAGAA	GNTCCCTCTTATGCTCTTGAAGTTCTT
Viruses used in Analysis	IMP181-6X	IMP181-3X-Conservative
	IMP181-3X-NonConservative	
	BFS932	

Initial validation of the PCR assay was performed using a WEEV infectious clone plasmids that had their sequences confirmed using conventional Sanger-based dideoxynucleotide sequencing methodology. Preparation of PCR amplicons for pyrosequencing was performed using a Qiagen Pyromark Q96 vacuum workstation. Pyrosequencing was run in SNP mode on the Pyromark ID using Pyromark Gold reagents.

### Virulence assays in syrian golden hamsters

Female Golden Syrian hamsters 5–6 weeks of age were purchased from Charles River (Wilmington, MA). Cohorts (n = 16 for each virus; n = 6 for mock) were infected IP with 4 log_10_ PFU. Hamsters were infected with individual virus strains (BFS932, IMP181, and IMP181-6X) in order to compare pathogenesis and virulence. Proper inoculum doses were verified by plaque assay. Body weights were measured each day with blood harvested alternatively every other day in each subject. On days 2 through 5, three hamsters from each group (one from mock) were perfused with PBS as previously described [58). Major organs were harvested and half of each organ, or the contralateral organ, were placed in 10% neutral buffered formalin and the remaining halves were placed in DMEM containing 5% FBS for measurement of viral load by plaque assay (amount of media was adjusted for each sample according to its weight to obtain a uniform limit of detection). Histopathological analysis and viral burden plaque assays were conducted as previously described [58].

### Statistical analysis

For virus competition experiments in both mosquitoes and house sparrows, relative replicative fitness values for viruses in each sample were analyzed according to w = (f_0_/i_0_), where w is relative fitness, f_0_ is the proportion of virus in the sample, and i_0_ is the proportion of virus in the inoculum. Relative fitnesses were evaluated graphically to determine consistency between replicates and pooled for analysis. The relative fitness values calculated for each virus in competition with IMP181 were analyzed by two-tailed Wilcoxon tests, testing the relative fitness values against a null value of 1 (no difference in proportion between inoculum and final proportion in the mosquito). This type of analysis is commonly used to statistically analyze competition assay experiments for viruses [59].

Differences in hamster weight change between groups was determined by fitting a linear mixed model using lme4 package for R. Hamster weights were designated as the dependent variable, with day post-infection, virus group, and their interaction term as the independent variables. Unique hamster identification numbers were included as a random effect to account for variation in weight change that was attributed to individual variation. The interaction between day post-infection and virus genotype was evaluated using the ‘emtrends’ function in the ‘emmeans’ package for R to make pairwise comparisons of weight change between virus groups, while correcting for multiple comparisons with the Tukey method [60, 61].

Log rank test was used to determine significant differences in the survival data. Briefly, survival curves were generated for each group and considered separately, using the Kaplan-Meier method and compared statistically using the log rank test.

Differences in blood viremias and viral loads in the hamster organs between virus groups were analyzed by first log(x+1) transforming the dependent variable (Viremia or Viral Load PFU/mL) and analyzing by ANOVA. Day post-exposure and virus group were included as independent variables. For blood viremia data, 3 repeated measurements were removed prior to analysis. Significant effects for by virus group were analyzed by post-hoc Tukey’s Honest Significant Difference method to make pairwise comparisons between virus groups, correcting for multiple comparisons using the Holm’s procedure.

### Ethics statement

Work with Syrian Golden Hamsters was approved by UTMB’s IACUC under protocol 0209068B. Hamsters were anesthetized using isoflurane and sacrificed via perfusion. Work with House Sparrows was approved by CSU’s IACUC under protocol 16-6420A. House Sparrows were euthanized at the end of the study with carbon dioxide. All studies were conducted according to the NIH guidelines on the care and use of laboratory animals. Additionally, both universities are AAALAC accredited and our studies were conducted according to those guidelines as well. House Sparrows were caught in Larimer County, Colorado. State or federal permits are not required to catch House Sparrows.

## Supporting information

S1 Fig Mosquito salivary gland infection rates for competition assays. WEEV infection rates in the salivary glands. Day 3 n = 5 per replicate, day 5 n = 5 per replicate, day 10 n = 5 per replicate. Error bars indicate standard error. No groups were significantly different from one another on each day as determined by a one-way ANOVA with Tukey post-tests.
(TIF)

S2 Fig Viral burden in 5–6 week old Syrian golden hamsters that do not show significant differences following infection with IMP181, IMP181-6X, and BFS932. Panels show viral burden in the A) spleen, B) lung, and C) liver. No statistical significance was detected between groups.
(TIF)

S3 Fig Histopathology of the brain during peak disease in BFS932 infected 5–6 week old Syrian golden hamsters. Images taken at 20X. Images indicate: perivascular cuffing, inflammation & nuclear dust, and hemorrhage, marked arrows. Images were taken at day 5 post-infection.
(TIF)

S4 Fig Histopathology of the heart during peak disease in 5–6 week old Syrian golden hamsters. Heart images taken at 20X. Yellow arrows indicate foci of myocarditis. BFS932 image is from day 4 post-infection. IMP181-6X, IMP181, and MOCK images were taken at day 5 post-infection.
(TIF)

## References

[1] Epidemiological Alert: Zika virus infection 7 May 2015 [press release]. World Health Oranization2015.

[2] Weaver SC, Costa F, Garcia-Blanco MA, Ko AI, Ribeiro GS, Saade G, et al Zika virus: History, emergence, biology, and prospects for control. Antiviral Research. 2016;130:69–80. 10.1016/j.antiviral.2016.03.010 26996139 PMC4851879

[3] Leparc-Goffart I, Nougairede A, Cassadou S, Prat C, de Lamballerie X. Chikungunya in the Americas. The Lancet. 2014;383(9916):514. 10.1016/S0140-6736(14)60185-9 24506907

[4] Family—Togaviridae In: King AMQ, Adams MJ, Carstens EB, Lefkowitz EJ, editors. Virus Taxonomy. 9th ed San Diego: Elsevier; 2012 p. 1103–10.

[5] Strauss JH, Strauss EG. The alphaviruses: gene expression, replication, and evolution. Microbiological Reviews. 1994;58(3):491–562. 7968923 10.1128/mr.58.3.491-562.1994 PMC372977

[6] Snyder JE, Kulcsar KA, Schultz KLW, Riley CP, Neary JT, Marr S, et al Functional Characterization of the Alphavirus TF Protein. Journal of Virology. 2013;87(15):8511–23. 10.1128/JVI.00449-13 23720714 PMC3719798

[7] Bergren NA, Auguste AJ, Forrester NL, Negi SS, Braun WA, Weaver SC. Western Equine Encephalitis Virus: Evolutionary Analysis of a Declining Alphavirus Based on Complete Genome Sequences. Journal of Virology. 2014;88(16):9260–7. 10.1128/JVI.01463-14 24899192 PMC4136285

[8] Weaver SC, Kang W, Shirako Y, Rumenapf T, Strauss EG, Strauss JH. Recombinational history and molecular evolution of western equine encephalomyelitis complex alphaviruses. Journal of Virology. 1997;71(1):613–23. 8985391 10.1128/jvi.71.1.613-623.1997 PMC191092

[9] Reisen WK, Monath TP. Western equine encephalomyelitis In: Monath TP, editor. The Arboviruses: Epidemiology and Ecology. V. Boca Raton, FL: CRC Press; 1988 p. 89–137.

[10] Hardy JL. The Ecology of Western Equine Encephalomyelitis Virus in the Central Valley of California, 1945–1985. The American Journal of Tropical Medicine and Hygiene. 1987;37(3 Part 2):18S–32S. 3318522 10.4269/ajtmh.1987.37.18s

[11] Bowers JH, Hayes RO, Hughes TB. Studies on the role of mammals in the natural history of western encephalitis in Hale County, Texas. Journal of Medical Entomology. 1966;6(175). 10.1093/jmedent/6.2.175 5817501

[12] Hardy JL, Milby MM, Wright ME, Beck AJ, Presser SB, Bruen JP. Natural and experimental arboviral infections in a population of blacktail jackrabbits along the Sacramento River in Butte County, California (1971–1974). Journal of Wildlife Diseases. 1977;13(383). 10.7589/0090-3558-13.4.383 24228958

[13] Hardy JL, Reeves WC, Rush WA, Nir YD. Experimental Infection with Western Equine Encephalomyelitis Virus in Wild Rodents Indigenous to Kern County, California. Infection and Immunity. 1974;10(3):553–64. 4426699 10.1128/iai.10.3.553-564.1974 PMC422990

[14] Aviles G, Sabattini MS, Mitchell CJ. Transmission of Western Equine Encephalomyelitis Virus by Argentine Aedes albifasciatus (Diptera: Culicidae). Journal of Medical Entomology. 1992;29(5):850–3. 10.1093/jmedent/29.5.850 1404265

[15] Calisher CH, Monath TP, Mitchell CJ, Sabattini MS, Cropp CB, Kerschner J, et al Arbovirus Investigations in Argentina, 1977–1980: III. Identification and Characterization of Viruses Isolated, Including New Subtypes of Western and Venezuelan Equine Encephalitis Viruses and Four New Bunyaviruses (Las Maloyas, Resistencia, Barranqueras, and Antequera). The American Journal of Tropical Medicine and Hygiene. 1985;34(5):956–65. 2863990

[16] Meyer KF, Haring CM, Howitt B. The etiology of epizootic encephalomyelitis of horses in the San Joaquin Valley, 1930. Science. 1931;74(1913):227–8. 10.1126/science.74.1913.227 17834966

[17] Sponseller ML, Binn LN, W L. W, Yager RH. Field Strains of Western Encephalitis Virus in Ponies: Virologic, Clinical, and Pathologic Observations. American Journal of Veterinary Research. 1966;27(121):1591–8. 5971613

[18] Byrne RJ, French GR, Yancey FS, Gochenour WS, Russell PK, Ramsburg HH, et al Clinical and Immunological interrelationships among Venezuelan, eastern, and western encephalomyelitis in burros. American Journal of Veterinary Research. 1964;25(24):24–31. 14103234

[19] Giltner LT, Shahan MS. The present Status of infectious Equine Encephalomyelitis in the United States. Journal of the American Veterinary Medical Association. 1936;88(3):363–74. 21005494

[20] Medovy H. Western equine encephalomyelitis in infants. Journal of Pediatrics. 1943;22(308).

[21] Minke JM, Audonnet J-C, Fischer L. Equine viral vaccines: the past, present and future. Vet Res. 2004;35(4):425–43. 10.1051/vetres:2004019 15236675

[22] CDC. Western Equine Encephalitis virus Neuroinvasive Disease Cases* Reported by State, 1964–2010. CDC; 2010.

[23] Neufeld J, Nayar G. Western equine encephalitis in Manitoba-equine cases, 1975–1981 In: Sekla L, editor. Western Equine Encephalitis in Manioba. Winnipeg: Ministry of Health; 1982 p. 62.

[24] Sekla L, Gadawski R, Nayar G, Brust R. A compilation of data on arboviruses surveillance in manitoba: 1975–1991. Proceedings of the Entomological Society of Manitoba. 1991;47:30–43.

[25] Reisen WK, Wheeler SS. Surveys for Antibodies Against Mosquitoborne Encephalitis Viruses in California Birds, 1996–2013. Vector-Borne and Zoonotic Diseases. 2016;16(4):264–82. 10.1089/vbz.2015.1888 26974395 PMC4800269

[26] Disease Maps [Internet]. Centers for Disease Control and Prevention. 2016 [cited May 23, 2016]. http://diseasemaps.usgs.gov/mapviewer/.

[27] Robb LL, Hartman DA, Rice L, deMaria J, Bergren NA, Borland EM, et al. Continued Evidence of Decline in the Enzootic Activity of Western Equine Encephalitis Virus in Colorado. 2018. 10.1093/jme/tjy214 30535264

[28] Forrester NL, Kenney JL, Deardorff E, Wang E, Weaver SC. Western Equine Encephalitis submergence: Lack of evidence for a decline in virus virulence. Virology. 2008;380(2):170–2. 10.1016/j.virol.2008.08.012 18801549 PMC2574696

[29] Logue CH, Bosio CF, Welte T, Keene KM, Ledermann JP, Phillips A, et al Virulence variation among isolates of western equine encephalitis virus in an outbred mouse model. Journal of General Virology. 2009;90(8):1848–58. 19403754 10.1099/vir.0.008656-0 PMC2887574

[30] Mossel EC, Ledermann JP, Phillips AT, Borland EM, Powers AM, Olson KE. Molecular determinants of mouse neurovirulence and mosquito infection for Western equine encephalitis virus. PLoS One. 2013;8(3):e60427 10.1371/journal.pone.0060427 23544138 PMC3609757

[31] Reisen WK, Fang Y, Brault AC. Limited Interdecadal Variation in Mosquito (Diptera: Culicidae) and Avian Host Competence for Western Equine Encephalomyelitis Virus (Togaviridae: Alphavirus). The American Journal of Tropical Medicine and Hygiene. 2008;78(4):681–6. 18385369

[32] Losos JB, Baum DA, Futuyma DJ, Hoekstra HE, Lenski RE, Moore AJ, et al The Princeton Guide to Evolution. Princeton, UNITED STATES: Princeton University Press; 2013.

[33] Duarte E, Clarke D, Moya A, Domingo E, Holland J. Rapid fitness losses in mammalian RNA virus clones due to Muller’s ratchet. Proceedings of the National Academy of Sciences. 1992;89(13):6015–9. 10.1073/pnas.89.13.6015 PMC402129 1321432

[34] Holland JJ, de la Torre JC, Clarke DK, Duarte E. Quantitation of relative fitness and great adaptability of clonal populations of RNA viruses. Journal of Virology. 1991;65(6):2960–7. 2033662 10.1128/jvi.65.6.2960-2967.1991 PMC240937

[35] Martínez MA, Carrillo C, González-Candelas F, Moya A, Domingo E, Sobrino F. Fitness alteration of foot-and-mouth disease virus mutants: measurement of adaptability of viral quasispecies. Journal of Virology. 1991;65(7):3954–7. 1645804 10.1128/jvi.65.7.3954-3957.1991 PMC241436

[36] Bellamy R, Kardos E. A strain of Culex tarsalis Coq. reproducing without blood meals. Mosq News. 1958;18:132–4.

[37] Quiñones-Mateu ME, Ball SC, Marozsan AJ, Torre VS, Albright JL, Vanham G, et al A Dual Infection/Competition Assay Shows a Correlation between Ex Vivo Human Immunodeficiency Virus Type 1 Fitness and Disease Progression. Journal of Virology. 2000;74(19):9222–33. 10.1128/jvi.74.19.9222-9233.2000 10982369 PMC102121

[38] Weaver SC, Brault AC, Kang W, Holland JJ. Genetic and Fitness Changes Accompanying Adaptation of an Arbovirus to Vertebrate and Invertebrate Cells. Journal of Virology. 1999;73(5):4316–26. 10196330 10.1128/jvi.73.5.4316-4326.1999 PMC104213

[39] Wu H, Huang Y, Dykes C, Liu D, Ma J, Perelson AS, et al Modeling and Estimation of Replication Fitness of Human Immunodeficiency Virus Type 1 In Vitro Experiments by Using a Growth Competition Assay. Journal of Virology. 2006;80(5):2380–9. 10.1128/JVI.80.5.2380-2389.2006 16474144 PMC1395363

[40] Tsetsarkin KA, Vanlandingham DL, McGee CE, Higgs S. A Single Mutation in Chikungunya Virus Affects Vector Specificity and Epidemic Potential. PLoS Pathog. 2007;3(12):e201 10.1371/journal.ppat.0030201 18069894 PMC2134949

[41] Tsetsarkin KA, McGee CE, Volk SM, Vanlandingham DL, Weaver SC. Epistatic Roles of E2 Glycoprotein Mutations in Adaption of Chikungunya virus to *Ades Albopictus* and *Ae*. *Aegypti* Mosquitos. PLoS ONE. 2009;4(8):e6835 10.1371/journal.pone.0006835 19718263 PMC2729410

[42] Tsetsarkin K, Weaver SC. Sequential adaptive mutations enhance efficient vector switching by chikungunya virus and its epidemic emergence. PLoS Pathog. 2011;7(12):e1002412 10.1371/journal.ppat.1002412 22174678 PMC3234230

[43] Tsetsarkin K, Chen R, Yun R, Rossi SL, Plante K, Guerbois M, et al Multi-peaked adaptive landscape for chikungunya virus evolution predicts continued fitness optimization in Aedes albopictus mosquitos. Nature Comm. 2014;5(1):4084. 10.1038/ncomms5084 PMC7091890 24933611

[44] Heise MT, Simpson DA, Johnston RE. A Single Amino Acid Change in nsP1 Attenuates Neurovirulence of the Sindbis-Group Alphavirus S.A.AR86. Journal of Virology. 2000;74(9):4207 10.1128/jvi.74.9.4207-4213.2000 10756033 PMC111935

[45] Klimstra WB, Ryman KD, Johnston RE. Adaptation of Sindbis Virus to BHK Cells Selects for Use of Heparan Sulfate as an Attachment Receptor. Journal of Virology. 1998;72(9):7357–66. 9696832 10.1128/jvi.72.9.7357-7366.1998 PMC109960

[46] Dropulic LK, Hardwick JM, Griffin DE. A single amino acid change in the E2 glycoprotein of Sindbis virus confers neurovirulence by altering an early step of virus replication. Journal of virology. 1997;71(8):6100–5. 9223504 10.1128/jvi.71.8.6100-6105.1997 PMC191870

[47] Hardy JL, Reeves WC, Sjogren RD. Variation in the susceptibility of field and laboratory populations of *Culex tarsalis* to experimental infection with western equine encephalomyelitis virus. American Journal of Epidemiology. 1976;103(5):498–505. 10.1093/oxfordjournals.aje.a112251 58557

[48] Coffey LL, Vasilakis N, Brault AC, Powers AM, Tripet F, Weaver SC. Arbovirus evolution in vivo is constrained by host alternation. Proceedings of the National Academy of Sciences. 2008;105(19):6970–5. 10.1073/pnas.0712130105 PMC2383930 18458341

[49] Grubaugh ND, Weger-Lucarelli J, Murrieta RA, Fauver JR, Garcia-Luna SM, Prasad AN, et al Genetic Drift during Systemic Arbovirus Infection of Mosquito Vectors Leads to Decreased Relative Fitness during Host Switching. Cell Host Microbe. 2016;19(4):481–92. 10.1016/j.chom.2016.03.002 27049584 PMC4833525

[50] Jerzak GVS, Brown I, Shi P-Y, Kramer LD, Ebel GD. Genetic diversity and purifying selection in West Nile virus populations are maintained during host switching. Virology. 2008;374(2):256–60. 10.1016/j.virol.2008.02.032 18395240 PMC2409985

[51] Holmes EC. Patterns of Intra- and Interhost Nonsynonymous Variation Reveal Strong Purifying Selection in Dengue Virus. Journal of Virology. 2003;77(20):11296 10.1128/JVI.77.20.11296-11298.2003 14512579 PMC224983

[52] Kilby ER. The Demographics of the US Equine Population In: Salem DJ, Rowan AN, editors. The State of Animals IV: 2007. New York: Humane Society Press; 2007.

[53] Brault AC, Powers AM, Ortiz D, Estrada-Franco JG, Navarro-Lopez R, Weaver SC. Venezuelan equine encephalitis emergence: Enhanced vector infection from a single amino acid substitution in the envelope glycoprotein. Proceedings of the National Academy of Sciences of the United States of America. 2004;101(31):11344–9. 10.1073/pnas.0402905101 15277679 PMC509205

[54] Anishchenko M, Bowen RA, Paessler S, Austgen L, Greene IP, Weaver SC. Venezuelan encephalitis emergence mediated by a phylogenetically predicted viral mutation. Proceedings of the National Academy of Sciences of the United States of America. 2006;103(13):4994–9. 10.1073/pnas.0509961103 16549790 PMC1458783

[55] Gorchakov R, Wang E, Leal G, Forrester NL, Plante K, Rossi SL, et al Attenuation of Chikungunya Virus Vaccine Strain 181/Clone 25 Is Determined by Two Amino Acid Substitutions in the E2 Envelope Glycoprotein. Journal of Virology. 2012;86(11):6084–96. 10.1128/JVI.06449-11 22457519 PMC3372191

[56] Lidbury BA, Rulli NE, Musso CM, Cossetto SB, Zaid A, Suhrbier A, et al Identification and Characterization of a Ross River Virus Variant That Grows Persistently in Macrophages, Shows Altered Disease Kinetics in a Mouse Model, and Exhibits Resistance to Type I Interferon. Journal of Virology. 2011;85(11):5651–63. 10.1128/JVI.01189-10 21430046 PMC3094974

[57] Beaty B, Calisher C, Shope R. Arboviruses. Diagnostic procedures for viral, rickettsial and chlamydial infections 6th ed Washington: American Public Health Association 1989:797–856.

[58] Plante KS, Rossi SL, Bergren NA, Seymour RL, Weaver SC. Extended Preclinical Safety, Efficacy and Stability Testing of a Live-attenuated Chikungunya Vaccine Candidate. PLoS Neglected Tropical Diseases. 2015;9(9):e0004007 10.1371/journal.pntd.0004007 26340754 PMC4560411

[59] Gandhi RT, Zheng L, Bosch RJ, Chan ES, Margolis DM, Read S, et al The effect of raltegravir intensification on low-level residual viremia in HIV-infected patients on antiretroviral therapy: a randomized controlled trial. PLoS Med [Internet]. 2010 2010/08//; 7(8). Available from: http://europepmc.org/abstract/MED/20711481 http://europepmc.org/articles/PMC2919424?pdf=render http://europepmc.org/articles/PMC2919424 https://www.ncbi.nlm.nih.gov/pmc/articles/pmid/20711481/pdf/?tool=EBI https://www.ncbi.nlm.nih.gov/pmc/articles/pmid/20711481/?tool=EBI 10.1371/journal.pmed.1000321. PMC2919424 20711481

[60] Bates D, Mächler M, Bolker B, Walker S. Fitting Linear Mixed-Effects Models Using lme4. Journal of Statistical Software; Vol 1, Issue 1 (2015). 2015.

[61] Lenth R, Singmann H, Love J, Buerkner P, Herve M. emmeans: Estimated Marginal Means, aka Least-Squares Means. In: Lenth R, editor. 1.4 ed2019.

