# Supplementary figures and images for: “Submergence” of Western equine encephalitis virus: Evidence of positive selection argues against genetic drift and fitness reductions
Source: PLoS Pathog. 2020 Feb 6;16(2):e1008102. doi: 10.1371/journal.ppat.1008102 (PMC7029877; doi:10.1371/journal.ppat.1008102)

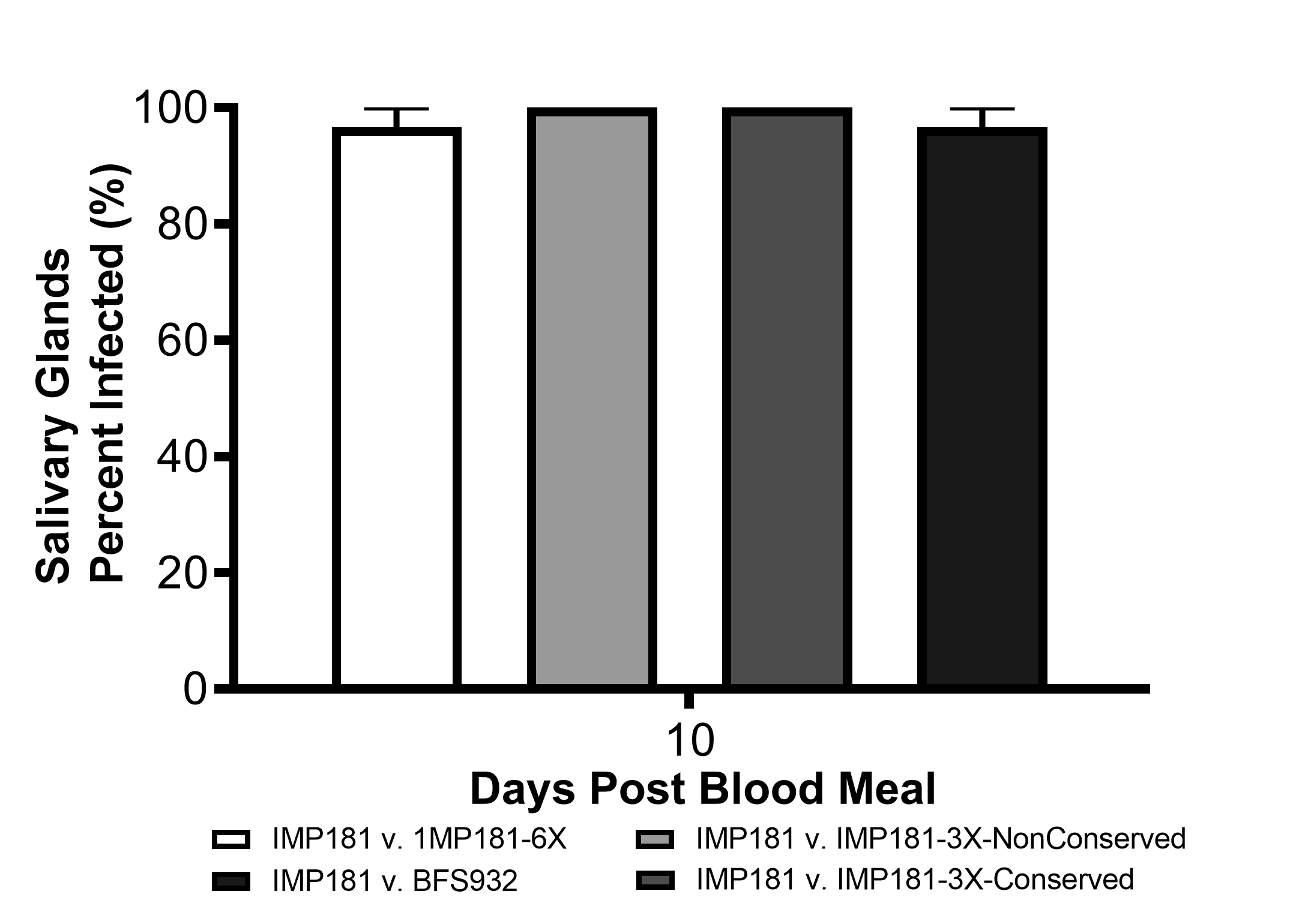

Supplement: S1 Fig — WEEV infection rates in the salivary glands. Day 3 n = 5 per replicate, day 5 n = 5 per replicate, day 10 n = 5 per replicate. Error bars indicate standard error. No groups were significantly different from one another on each day as determined by a one-way ANOVA with Tukey post-tests. (TIF) [file ppat.1008102.s001.tif]

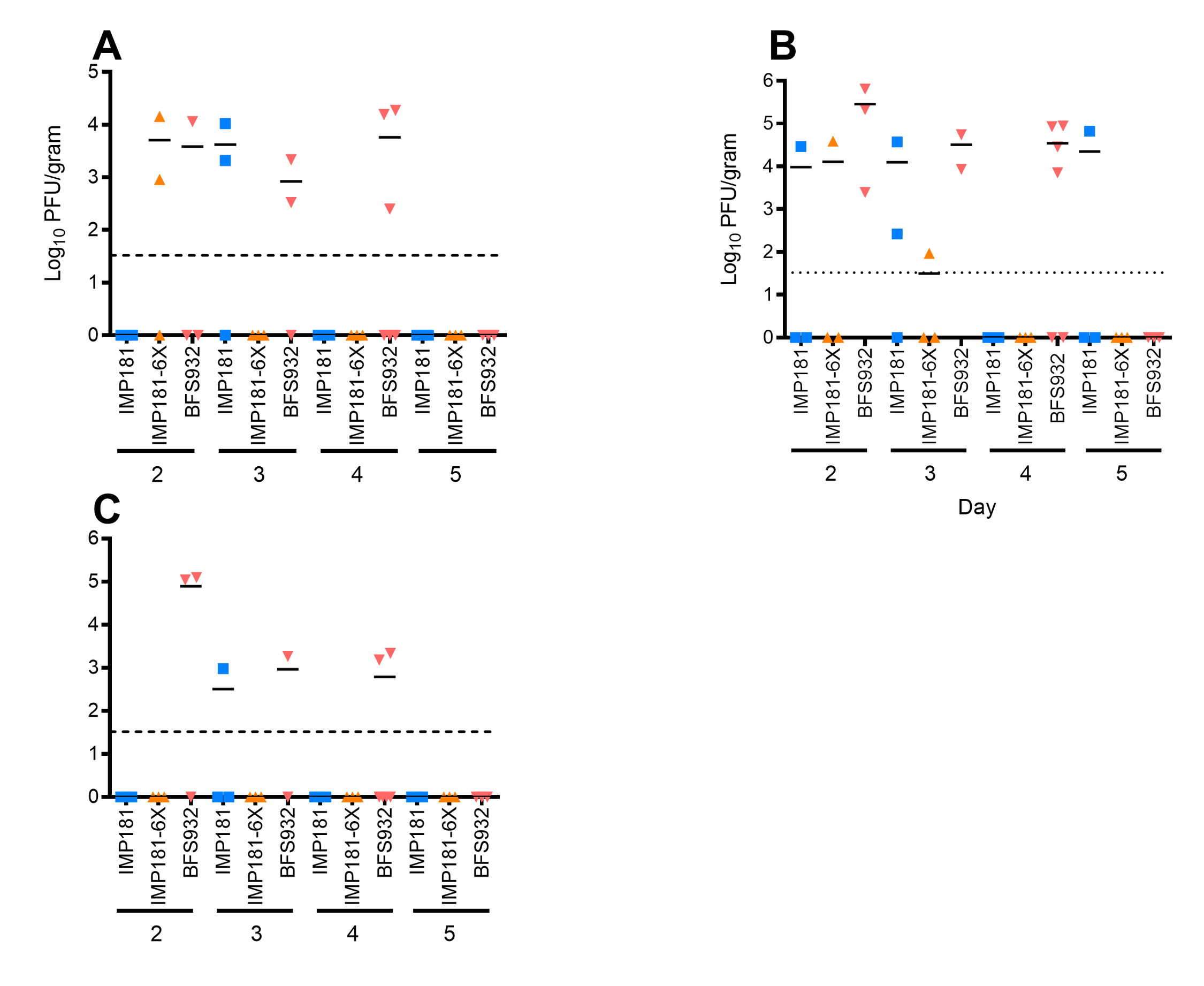

Supplement: S2 Fig — Panels show viral burden in the A) spleen, B) lung, and C) liver. No statistical significance was detected between groups. (TIF) [file ppat.1008102.s002.tif]

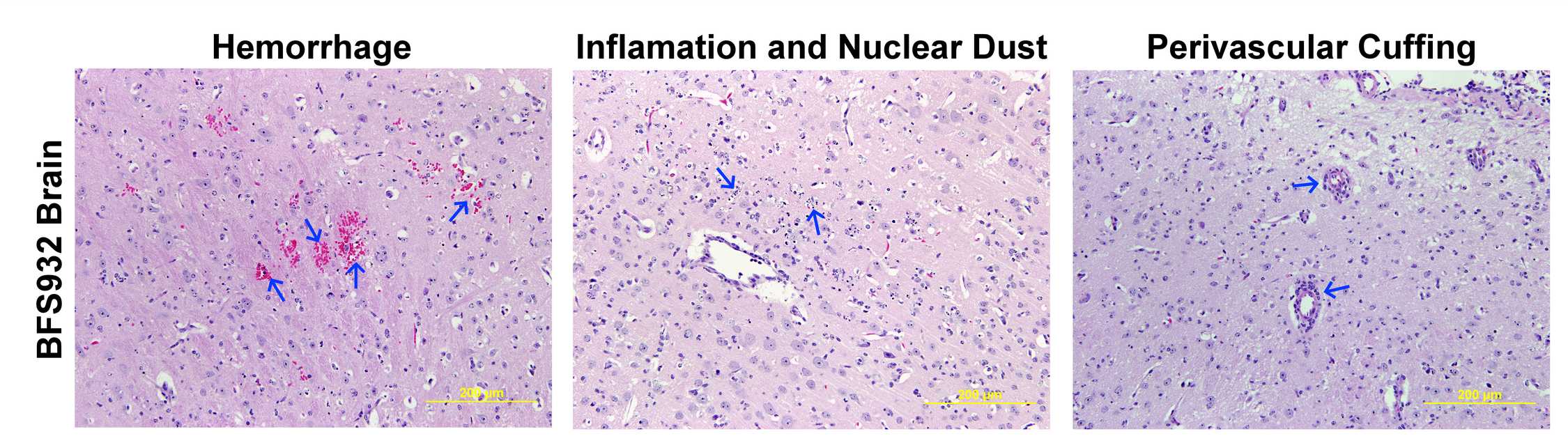

Supplement: S3 Fig — Images taken at 20X. Images indicate: perivascular cuffing, inflammation & nuclear dust, and hemorrhage, marked arrows. Images were taken at day 5 post-infection. (TIF) [file ppat.1008102.s003.tif]

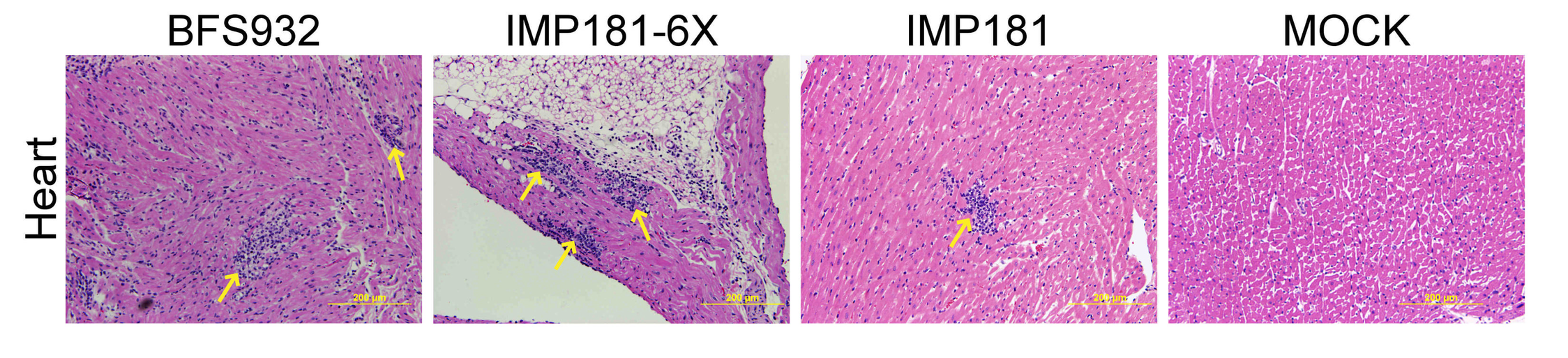

Supplement: S4 Fig — Heart images taken at 20X. Yellow arrows indicate foci of myocarditis. BFS932 image is from day 4 post-infection. IMP181-6X, IMP181, and MOCK images were taken at day 5 post-infection. (TIF) [file ppat.1008102.s004.tif]
